# Next‐generation disease modeling with direct conversion: a new path to old neurons

**DOI:** 10.1002/1873-3468.13678

**Published:** 2019-11-26

**Authors:** Larissa Traxler, Frank Edenhofer, Jerome Mertens

**Affiliations:** ^1^ Department of Genomics Stem Cell Biology & Regenerative Medicine Institute of Molecular Biology & CMBI Leopold‐Franzens‐University Innsbruck Innsbruck Austria; ^2^ Laboratory of Genetics The Salk Institute for Biological Studies La Jolla CA USA

**Keywords:** aging, cellular reprogramming, direct conversion, disease modeling, epigenetics, geriatric diseases, induced neurons, metabolism, neurodegenerative disorders

## Abstract

Within just over a decade, human reprogramming‐based disease modeling has developed from a rather outlandish idea into an essential part of disease research. While iPSCs are a valuable tool for modeling developmental and monogenetic disorders, their rejuvenated identity poses limitations for modeling age‐associated diseases. Direct cell‐type conversion of fibroblasts into induced neurons (iNs) circumvents rejuvenation and preserves hallmarks of cellular aging. iNs are thus advantageous for modeling diseases that possess strong age‐related and epigenetic contributions and can complement iPSC‐based strategies for disease modeling. In this review, we provide an overview of the state of the art of direct iN conversion and describe the key epigenetic, transcriptomic, and metabolic changes that occur in converting fibroblasts. Furthermore, we summarize new insights into this fascinating process, particularly focusing on the rapidly changing criteria used to define and characterize *in vitro*‐born human neurons. Finally, we discuss the unique features that distinguish iNs from other reprogramming‐based neuronal cell models and how iNs are relevant to disease modeling.

## Graphical abstract

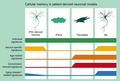

## Abbreviations

**ALS**, amyotrophic lateral sclerosis

**HD**, Huntington’s disease

**iNs**, induced neurons

**iPSCs**, induced pluripotent stem cells

**TF**, transcription factor

## Why we need human neurons in a dish

The immense processing power of the human brain is enabled by unique features of the brain’s hardware: around 86 billion neurons with each one of them possessing thousands of synaptic connections 1. It is believed that molecular and cell biological insults to neurons lead to hardware issues and ultimately manifest as neurological disorders. Animal studies, both *in vivo* and *in vitro*, have provided eye‐opening insights into the inner workings of neurons and the brain. Animal models of brain disorders have however been found to not necessarily reflect complex human conditions and have unfortunately not been very predictive for the evaluation of drug candidates for several diseases. Alzheimer’s disease stands out as a prime example to illustrate this puzzle. Animals, typically mice, can be genetically engineered to reflect central pathological hallmarks of the human disease, but all drug candidates that were developed based on successful animal studies have failed in clinical trials 2, 3. Animal models for other neurodegenerative and neuropsychiatric disorders all face their own individual but similar challenges. This dilemma has promoted the view that human neurons possess their unique biology that might be vital for studying aspects of many diseases, but the inaccessibility of human brain tissue renders it near‐impossible to functionally and molecularly study neurons directly in the human brain. Driven by the idea that human neurons *in vitro* can help to capture and better understand disease‐related factors that require a human neuronal cell physiology, human genetics, epigenetic signatures, and age, the generation of human neurons as disease models has received broad attention as a potential game‐changer.

Notably, the direct conversion from one cell type into another, often also called fate conversion, direct reprogramming, or transdifferentiation, has been first demonstrated already in the 1980s, where it was shown that overexpression of the transcription factor (TF) MyoD can convert fibroblasts into myoblast‐like cells 4. Interestingly, only 30 years later and a few years after the invention of induced pluripotent stem cells (iPSCs) 5, the direct conversion of fibroblasts into induced neurons (iNs) was discovered 6. With the invention of iNs, direct conversion strategies, also for other cell types, regained broader interest. From this point onward, direct conversion technologies have grown rapidly, and are today mostly regarded as a subdiscipline of the stem cell field, where they are seen as alternative approaches to generate cell types of interest from human patients and donors for disease modeling or regenerative purposes 7, 8. This boom in applications can be mostly attributed to the explosion of new technologies tailored to iPSC‐based systems, most of which are also suitable for directly converted cells. These technologies encompass tools and strategies to harness human donor/patient‐specific cells for basic human biology research 9, 10, 11, 12, disease modeling 13, 14, 15, 16, 17, drug development and safety 18, 19, 20, 21, or cell replacement strategies 22. Although on first sight iNs might appear as ‘just another way’ to generate neurons in the dish, there are important technical and conceptual differences between iPSC‐derived neurons and iNs to be noted. While some of these differential properties cause limitations of the iN technology for certain applications, some properties uniquely qualify iNs to address yet unmet needs. Here, we will review conversion strategies for human somatic cells into iNs, describe mechanistic insights and roadblocks to direct conversion, and discuss current standards and new criteria on how to characterize human neurons. We will further pay particular attention to the conceptual differences between iN conversion and other reprogramming methods and will highlight unique properties that set iNs apart for specific basic and translational applications.

## Enabling iN conversion

**Figure 1:**
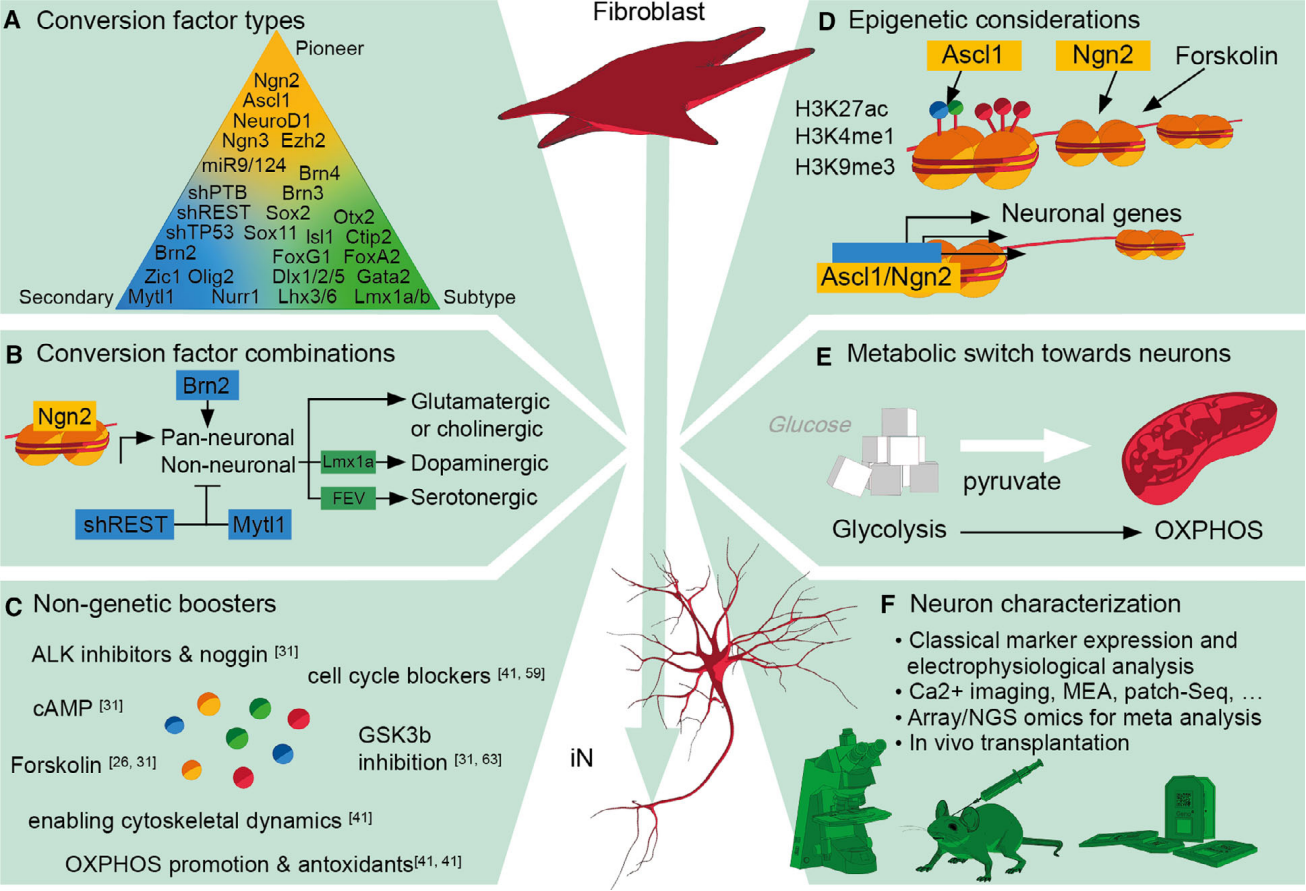
(A) Factors for direct iN conversion, including transcription factors (TFs), can be classified into pioneer and secondary factors. The TFs Ascl1 and Ngn2 are the two most widely used pioneer factors that can facilitate iN conversion on their own. Secondary factors do not induce conversion on their own and are instead used to achieve increased efficiencies and neuronal qualities. Myt1l is a prime example for a secondary TF, while miR‐9/124 and shRNAs against REST or PTB have neuron‐inducing capabilities and can be regarded as ‘in‐between’ pioneer and secondary factors. As pioneer factors typically do not (strongly) dictate subtype identity, subtype‐specific secondary factors can be added to induce a desired neuronal subtype. Some factors primarily regarded as subtype‐specifiers, such as Nurr1, Sox11, and Brn3/4, also display considerable iN boosting efficiencies. (B) First, a pioneer factor induces a broad neuronal transcriptional program, a process that benefits from secondary factors that can help induce a neuronal program either by transactivation activity (e.g., Brn2) or by repressing the non‐neuronal program (e.g., Myt1l and REST inhibition). Once a broad epigenetic neuronal context is established, subtype‐specific secondary factors (e.g., Lmx1a and FEV) can direct iN toward specific epigenetically stable subtype identities. (C) Nongenetic boosters of iN conversion are used to increase efficiencies and to obtain iNs with better neuronal qualities faster. Typically, chemical boosters are small molecules that block or activate signaling pathways involved in direct conversion or that are known to benefit neuronal differentiation, maturation, or survival. (D) Pioneer TFs can bind and open up closed chromatin regions that are essential to initiate and jump‐start iN conversion. However, even pioneer TFs require specific epigenetic marks in order to bind closed chromatin (e.g., trivalent state for Ascl1), and iN boosters (e.g., forskolin) have been found to be directly involved in chromatin remodeling to permit more efficient iN conversion. (E) A radical metabolic switch from glycolysis (the primary source of energy for stem cells and fibroblasts) toward mitochondria‐based oxidative phosphorylation (OXPHOS) is a major obstacle for neuronal conversion and iN survival. Enzymatic activity of LDHA (pyruvate to lactate) and reactive oxygen species (a side product of OXPHOS) prohibits iN conversion, whereas promotion of OXPHOS and antioxidant activity enhances iN conversion. (F) Neuronal identity can be assessed using neuronal marker expression or electrophysiological properties, but can be further characterized more profoundly with next‐generation transcriptomic, epigenetic, or metabolic analyses.

Unlike neural differentiation protocols starting from iPSCs, direct iN conversion does not follow the concerted chronological stages of development, as one cell type is rather directly transformed into another one 23. Overexpression of transcription factors (TFs) driving iN conversion (hereafter referred to as conversion TFs) overrides the cell type‐specific transcriptional profile of the starting population and instantly activates a neuronal transcriptional program (with a few exceptions), permitting cell‐type changes in a very short time 24. The TFs bind to regulatory elements in the starting cells’ genome and jump‐start neuronal gene expression. In contrast to stem and progenitor cells, fully differentiated somatic cells possess a tightly regulated epigenetic landscape, with regions specific for other cell types inaccessible for most TFs. Conversion TFs that are sufficient for neuron induction stand out by their ability to bind to largely inaccessible ‘neuronal regions’ of the genome in differentiated non‐neuronal cell types. This ability classifies these factors as pioneer TFs (Fig. 1A); the list of known iN pioneer TFs currently includes Ascl1, Ngn2, and NeuroD1 25, 26, 27, 28. Although every starting cell type has a unique epigenetic landscape, they all have in common that their chromatin surrounding neuronal gene loci is closed, and a general rule is that most iN strategies involve at least one pioneer TF to access these closed regions. Fact‐checking supports the validity of this rule as (a) the vast majority of efficient iN protocols involve at least one pioneer factor (Table 1) 29, 30, (b) Ngn2 alone can convert up to 90% of human fibroblasts into iNs, and (c) also Ascl1 alone can induce neuron‐like cells from fibroblasts 6, 26, 31. Pioneer TFs induce the expression of endogenous secondary pro‐neuronal TFs or of factors that repress the starting cell type‐specific transcriptome, which further contributes to establishing neuronal identity 27. Chromatin accessibility and transcriptome data have suggested that Zfp238, Sox8, and Dlx3 are among the most important endogenous secondary TF genes downstream of Ascl1 32. Some data indicate that Ngn2, when using an appropriate conversion medium, not only binds most of the Ascl1 binding sites in fibroblasts, but also possesses many additional binding sites 30, 33. However, other data suggest that Ascl1 and Ngn2 possess divergent binding patterns that result in distinct chromatin states and different neuronal fates 34. While further (meta‐)analysis will likely shed more light on these different views, it is not surprising that the most efficient and reliable conversion strategies involve the combined expression of Ascl1 and Ngn2 9, 10, 31, 35. Recently, it has been suggested that a huge variety of TF combinations can be applied to generate subtype‐specific iNs from fibroblasts (Table 1), and TF screening studies for iN conversion have led to the identification of additional pro‐neuronal factors, such as Brn3a/b/c, Brn4s, and Ezh2 36, 37. Interestingly, differences in TF choice were noted between species, as, for example, NeuroD1 and Ngn2 were used predominantly in human iN protocols and not in rodent protocols, but no mouse‐specific or human‐specific TF combinations have been established thus far 6, 25. Further, in some iN studies, the age of the human donor has been negatively correlated with the percentage of iNs obtained 38, and fibroblasts from adult human donors are resistant to Ascl1/Brn2‐based conversion, while fetal fibroblasts are highly amenable 38. RE1‐silencing complex (REST), a major neuronal gene repressor in non‐neuronal cells, and the aging‐associated TF FOXO3 play important roles in controlling neuronal gene expression and show differential activity between fetal and adult/old fibroblasts, resulting in decreased conversion efficacy in aged starting cells 38, 39, 40. Again, the combination of the two pioneer factors Ascl1 and Ngn2, for example, fused via a 2A peptide sequence, has yielded iN efficiencies of typically over 50% across large sample sizes and does not appear to be affected by donor age 9, 10, 35. In this setting, however, the presence of a cocktail of small‐molecular iN boosters might further mask an age‐related inhibitory effect 31, 41.

**Table 1:** Direct neuronal conversion strategies.

Cell source	Species	Conversion strategy	Specification	Citation
Fibroblasts	Mouse	**Ascl1**, Brn2, Myt1l (BAM)	First direct conversion from fibroblasts	6
		**Ascl1** +/− Brn2, Myt1l		24, 32, 38, 48, 49, 51
		B**A**M	Mesoporous silica nanoparticles, dopaminergic	174
		B**A**M	CRISPR‐based	59
		B**A**M	Electroporation, 3D	175
		Small molecules		15, 176
		**Ngn2**		33
		**Ascl1**, Foxg1, Sox2, Dlx5, Lhx6	GABAergic	83
Fibroblasts	Human	B**A**M + **NEUROD1**	First direct conversion from human fibroblasts	25
		B**A**M	First direct conversion from adult human fibroblasts	177
		**ASCL1, NGN2**		9, 10, 31, 34, 41
		**ASCL1, NGN2**, NKX2.2, FEV, GATA2, LMX1B	Serotonergic	35
		**ASCL1**, FOXA2, LMX1B, FEV	Serotonergic	75
		**ASCL1**, NURR1, LMX1A	Dopaminergic	178, 179
		**ASCL1**, PHOX2B, PHOX2A, AP‐2A, GATA3, HAND2, NURR1	Noradrenergic	180
		**ASCL1**, KD of P53, HEY2, and PRPX2		181
		**NGN2**/1, BRN3	Sensory neurons	76
		Small molecules		15, 182
		BRN2, MYT1L, FEZF2	Cortical neurons	81
		miR‐9/9*‐124, BCL11B/CTIP2, DLX1, DLX2, MYT1L	Striatal neurons	7, 57
		miR‐9/9*‐124 +/− B**A**M		44, 46, 47, 183
		miR‐9/9*‐124, ISL1, LHX3	Motor neurons	79
		PTB‐KD		42
Glia	Mouse	**NeuroD1**		27
		NeuroD2	*In vivo*	184
		Dlx2	GABAergic neurons	185
		**Ngn2**	*In vivo*	185
		**Ascl1**, Lmx1a, miR‐218	*In vivo*, parvalbumin interneurons	82
Astrocytes	Mouse	**Ascl1**	*In vitro*/*in vivo*	64, 186
		**Ascl1,** Lmx1b, Nurr1	Dopaminergic	179
		**Ascl1,** Phox2a, Phox2b, AP‐2a, Gata3, Hand2, Nurr1	Noradrenergic	181
		**Ascl1,** Sox2, Neurod1		187
		**Ngn2**	*In vivo*	113, 185
		**Ascl1, Ngn2**	Varying iN subtypes	73
		**NeuroD1**		80, 184
		Small molecules		188
	Human	**NEUROD1, ASCL1,** LMX1A, miR‐128	Dopaminergic neurons	80
		**ASCL1**	PTB‐mediated	189
		Small molecules	Fetal astrocytes	118, 190
		Small molecules	Adult astrocytes	191
Retina	Human	**NEUROD1,** PAX6, BRN2, MYT1L		192
Pericytes	Human	**ASCL1**, SOX2		69
		**ASCL1**, MYT1L, BRN2, TLX3, miR‐124	Cholinergic	193
T cells	Human	B**A**M + **NGN2**		91
Cord blood cells	Human	FOXM1, SOX2, MYC, SALL4, STAT6		194
Hepatocytes	Mouse	B**A**M		195
		Suz12, Ezh2, Meis1, Sry, Smarca4, Esr1, Pparg, Stat3		194

Bold font indicates pioneer transcription factors (also see Fig. 1A).

While pioneer TFs are sufficient for iN conversion, they are not essential and several laboratories have successfully obtained iNs without exploiting the classical pioneer TFs (Table 1). Those studies have harnessed the fact that neuronal genes are actively repressed in non‐neuronal cells and manipulated this mechanism. Activity of the ‘anti‐neuro’ REST complex is strongly supported by PTB proteins, and PTBs are key targets of the pro‐neuronal microRNAs miR‐9/9*‐124. The PTB‐REST‐miR‐9/9*‐124 ensemble is so powerful that mere interference with it, through shRNA‐mediated knockdown of PTBs or components of the REST complex or through overexpression of miR‐9/9*‐124, can replace pioneer transcription factors (Fig. 1A) 38, 42, 43, 44, 45. Similar to Ascl1 alone, also shRNA‐only and miRNA‐only iN protocols are quite inefficient and rely on several ‘helper’ factors (Fig. 1B) 31, 46, 47. For example, overexpression of Myt1l alone is insufficient to induce iNs from fibroblasts, but Myt1l remains one of the most valued iN ‘helpers’ in various protocols.

## Genetic iN boosters

Among the growing number of successful iN‐conversion protocols, two main strategies have crystalized that facilitate increased conversion efficiencies and more authentic neuronal outcomes: (a) co‐overexpression of pioneer TFs with boosting TFs, and (b) combination of pioneer TFs with media containing cocktails of signaling‐pathway modulators (Fig. 1C).

The first published direct conversion strategy was based on an overexpression of the three TFs, namely Ascl1, Brn2, and Myt1l (BAM factors), in mouse fibroblasts 6, which was then extended to BAM with NeuroD1 to convert human fibroblasts to iNs with a similar efficacy 25. Later studies elaborated on the first paper 10, where it was shown that Ascl1 alone is enough to mediate fibroblast‐to‐neuron conversion, whereas Brn2 and Myt1l boost conversion efficacy and neuron quality, but are unable to induce reprogramming on their own 31, 32, 48, 49. Myt1l is a master repressor of non‐neuronal genes and a prime example for a boosting TF (Fig. 1A,B) 50. Albeit lacking pioneer activity, co‐overexpression of Myt1l with pioneer TFs dramatically increases iN yields and improves functional properties of iNs in direct conversion protocols, especially those that otherwise fail to yield satisfactory neuronal numbers and properties 6, 25, 46. Myt1l can be regarded as the nemesis of the REST complex as it broadly represses non‐neuronal fates, such as the myogenic fate that has been identified as a common false diversion of converting cells on their way toward iNs 11, 48, 51. Unlike Myt1l, the boosting TF Brn2 acts as a classical activating secondary TF that binds to genomic regions that open in response to pioneer TFs 32. While not sufficient to induce neurons from fibroblasts, Brn2 alone can convert astrocytes into neuronal cells, indicating some pioneering properties (Fig. 1A) 52. In sharp contrast to pioneer and secondary TFs, mutations in Myt1l and Brn2 are associated with subtler neurological phenotypes linked to intellectual disability and neuropsychiatric conditions in humans 53, 54, 55, 56, whereas the pioneers Ascl1 and Ngn2 are well‐established master regulators of nervous system development, the mutation of which causes embryonic lethality.

Besides TFs, miRNAs have also been harnessed as iN boosters, and REST inhibition or overexpression of REST‐associated miRNAs could highly enhance conversion of adult and diseased fibroblasts in pioneer TF‐based settings 38, 57. Furthermore, in keeping with an active cell cycle being a major roadblock to iN‐conversion initiation, G1 arrest, for instance, achieved through high‐density contact inhibition, is beneficial for conversion initiation. Consistently, knocking down TP53 could enhance conversion efficacy by decreasing the number of fibroblasts in S phase at the start of neuronal induction 58. In general, identification and mechanistic characterization of boosting transgenes may not only yield important insights into the process of direct conversion, but also might help to engineer tailored conversion TFs and vectors in the near future 41, 59. However, co‐overexpression of multiple TFs is a technical challenge and always harbors the risk to induce bias. For example, TF activity might override important disease‐related signatures in iN models for neurological disorders 60. Conceivably, it is a general desire in the field to reduce the number of conversion TFs toward a minimal combination with the maximal effect. Driven by this idea, the alternative use of pathway modulators that repress the identity of the starting population and/or promote neuronal identity represents another strategy to boost iN efficiency and authenticity.

## Nongenetic iN boosters

Both purified and recombinant proteins and chemical compounds have been identified to enhance iN generation and have further been helpful to better understand the mechanisms of direct iN conversion (Fig. 1C). Soluble factors are often described to mechanistically ‘hit the same spot’ as conversion TFs, and thus can replace certain activities of the transgenes. First, and largely adopted from neural differentiation protocols, inhibition of TGF/ALK/SMAD signaling through the application of recombinant Noggin and small‐molecule ALK inhibitors, GSK3β inhibition, and cAMP/forskolin have been quickly identified to greatly enhance conversion yields and aid to obtain more authentic neurons (Fig. 1C) 26, 31, 61, 62. Interestingly, while TGF/ALK/SMAD and GSK3β inhibition is thought to destabilize non‐neuronal identities and promote neuronal fate‐stabilizing signaling 61, forskolin was shown to directly prime chromatin accessibility for more efficient Ngn2‐only conversion, a pioneer‐enhancing activity previously ascribed exclusively to TFs (Fig. 1D) 33. Subsequently, it has been shown that additional pathway modulations, including SIRT1 activation and HDAC inhibition, can be applied to increase the efficacy and maturity of the neuronal population obtained 62. Next, the cell cycle arrest‐promoting activity of Ascl1 and Ngn2 can be enhanced by knocking down *TP53*, serum withdrawal, CDK2 and mTOR inhibition, and chemical JAK/STAT inhibition, which further helps iN conversion by inhibiting both epithelial‐to‐mesenchymal transition and apoptosis 41, 58, 63. Enabling cytoskeletal dynamics through inhibition of integrin signaling facilitates the cell‐type switch 41. Inhibition of HIF‐1α improves iN conversion as it promotes the metabolic switch from glycolysis to mitochondria‐based oxidative phosphorylation, which is essential for neuronal identity 41, 64, 65. While nongenetic boosters are expected to be less invasive than transgenes, they often also possess very powerful neuroprotective functions and thus might, similar to TFs, mask important disease‐ and epigenetic age‐related signatures in iN‐based disease models 41.

## Subtype‐specific iN conversion

In the developing brain, as well as in iPSC differentiation, differentiating neural cells pass through a series of well‐defined developmental neural precursor stages, some of which are amenable to regionalizing patterning signals to specify specific neuronal subtypes 66. In direct iN conversion, these specialized precursor stages are instead skipped 48, 67. Thus, neuronal subtype specification cannot be easily achieved through the addition of patterning factors such as Wnt or Shh in the media, because the responsive cell type is not present at any time point during conversion. The most prominent conversion strategies using BAM, Ascl1/Ngn2, and miR‐9/9*‐124 typically give rise to a major population of excitatory glutamatergic neurons, and the Ngn2‐only protocols lead to excitatory cholinergic neurons (Fig. 1B) 25, 26, 31. While these observations somewhat imply a glutamatergic‐by‐default mechanism, or alternatively have led to the assumption that Ascl1 and Ngn2 are pro‐glutamatergic and pro‐cholinergic, respectively, reality appears less straightforward. For example, iNs generated from iPSCs through Ngn2‐only protocols are predominantly glutamatergic 16, 68. Ascl1/Sox2‐based iNs generated from pericytes *in vivo* resemble mixed GABAergic/glutamatergic cultures 69, and Ascl1 has been shown to induce oligodendroglial cells from adult neural stem cells in the dentate gyrus of mice 70. Also, both TFs are involved in midbrain and hindbrain neuronal differentiation and have varying capacities depending on the regional context 71, 72. Consistently, Ascl1/Ngn2‐based conversion of human fibroblasts into iNs leads to a major fraction of glutamatergic iNs, a smaller fraction of GABAergic iNs, and rare dopaminergic and serotonergic cells 10, 31, 35. Based on these observations, one might describe Ngn2 and Ascl1 as generally ‘pan‐neuronal’, with little subtype‐specifying activity (Fig. 1A). The epigenetic identity of the starting cell population and remnant signaling cues present during the fibroblast‐to‐iN transition state finally determine subtype identity 23, 34. In highly heterogeneous cell populations, such as astrocytes in the rodent brain or primary human fibroblasts, the subtype of the starting cell type was shown to determine subtype outcomes of the iNs 73. Both Ngn2 and Ascl1 seem to leave significant ‘wiggle room’ for subtype specification, which suggests addition of subtype‐specifying TFs to the mix for direct iN conversion into the neuronal cell type of interest (Fig. 1A,B). Depending on the combination of the pan‐neuronal pioneer TFs with subtype‐specific TFs, specific neuronal subtypes with distinct neurotransmitter and channel properties arise, providing a unique platform for studying specific cells 7, 36, 74. This concept has attracted attention beyond the crowd of reprogramming enthusiasts, as it allows generating specific neuronal subtypes that are specifically vulnerable, or resilient, to certain diseases. With the recent advances in subtype‐specific direct conversion, various disease‐specific neuronal subtypes have become available, such as medium spiny neurons for modeling Huntington’s disease (HD) 7, serotonergic neurons for modeling depressive and anxiety disorders 35, 75, sensory neurons for studying pain‐related diseases 76, motor neurons resembling an amyotrophic lateral sclerosis (ALS)‐related phenotype 77, 78, 79, dopaminergic neurons to model Parkinson’s disease 58, 62, 80, or cortical 81 and various types of interneurons that appear attractive to model, for example, Tourette’s syndrome or paroxysmal dystonia 82, 83. While these advances clearly offer novel tools to better understand neurological diseases, there is very strong evidence that classical neuronal diseases do not only affect neurons, but involve many cell types represented in the human brain 84. To meet this need to model the human neuron–glia cross talk *in vitro* with patient‐specific cells, astrocytes and oligodendrocytes are currently generated via direct conversion from human iPSCs, but direct conversion protocols from fibroblasts into induced astrocytes and oligodendrocytes have yet only been established for rodent cells 85, 86, 87, 88, 89. As an alternative route, direct conversion into induced neural stem cells, which then are amenable to directed ‘development‐mimicking’ differentiation, represents another way to generate glial cell types from human fibroblasts [reviewed in Erharter *et al*.]. Further, peripheral Schwann cells are of great interest for the study and treatment of spinal cord injuries and have been generated from adult human fibroblasts, representing exciting new possibilities for peripheral nerve regeneration 90. In conclusion, the number of protocols for neuronal subtype‐specific direct conversion literally exploded in the last couple of years, and we now have the means to generate a vast variety of neuronal subtypes *in vitro*. The next important steps in the near future would be the development of protocols for the generation of induced glial cell types also from human somatic cells and extension of existing protocols to other, more accessible starting populations, such as human peripheral blood cells 91.

## The road toward neurons: a steep and sloppy path?

The famous epigenetic landscape model established by Waddington is an outstanding model to describe cell fate switches during normal development and iPSC differentiation 30, 92, 93. Using this model, direct conversion has been depicted as a direct path from one valley to the other, straight over the highest mountains 93. However, given the apparent easiness of direct conversion and the fact that transition states appear unidirectional and with little developmental potency, a ‘tunnel’ metaphor might appear more useful. Also, the inherent emphasis on hierarchy in the Waddington model appears less suitable to describe direct conversion and further seems to fall short of describing the roles of stable (quiescent) intermediate and progenitor cell stages during development and plasticity of cell fate in response to external stimuli. As a result, adjusted metaphors such as a highly dynamic epigenetic landscape 94, the Cook island model 95, or the epigenetic disk model 96 have been introduced, all of which attempt to weaken the one‐way hierarchal character of the Waddington landscape. All these models have in common that cellular states correspond to valleys or holes, indicating that the epigenome favors certain stable states that correspond to cell types that exist *in vivo* at any given time, but rejects cellular chimeras, which would be cells that transiently share neuronal and starting cell properties, and which are rarely described 48, 67.

To explore the differences between the transcriptome path and outcome of stem cell differentiation and direct iN conversion, an elegant study employed single‐cell transcriptome analysis of neurons generated through neural stem cell differentiation and direct conversion of neural stem cells into iNs 67. Interestingly, iNs diverge from the differentiation path early on and do not follow the precise intermediate states of development, but generate a unique intermediate state that is unrelated to the donor and the target cells. However, after this ‘shortcut’, iNs appear to arrive at the same state as neurons obtained by differentiation, but without losing epigenetic information about age and disease.

Developmental and differentiation protocols are expected to roughly follow similar epigenetic sequences. Starting from somewhere between the zygote and blastocyst stages in development or from embryonic stem cells or iPSCs in differentiation protocols, modifications to the epigenetic landscape follow a hierarchical and highly orchestrated sequence of events that ultimately lead to a differentiated cell 97, 98. To initiate iN conversion however, the already defined and comparably inaccessible chromatin of the starting cell has to be targeted by an overexpressed pioneer TF capable of binding and activating closed chromatin regions 24, 28. Once chromatin remodeling is induced and the neuronal transcriptional program is started, non‐neuronal programs have to be suppressed. In contrast to the very time‐consuming and precisely timed process of differentiation, direct conversion induces a neuronal program around three to five days after transgene expression, which can lead to functional synapses already at around three weeks postinduction 6, 48. Converting cells from one germ layer to another, for instance, from mesoderm (fibroblasts) to ectoderm (neurons), requires substantial chromatin remodeling to make target‐cell‐type‐specific genes accessible to TFs. Such changes of cellular identity and the absence of a defined progenitor cell‐type stage indicate that iNs do not follow the typical developmental path from stem cell to postmitotic neuron, but rather represent a jump start of a then self‐propelled neuronal program 33, 41, 48, 91, 99. In recent years, several studies performing transcriptomic and epigenomic analysis on converting neurons gave rise to new insights and concepts that characterize fibroblast‐to‐neuron conversion. It has been found that pioneer TFs can be classified as either off‐target or on‐target TFs. Off‐target TFs, such as the Yamanaka factors during iPSC reprogramming, initially bind to target sites in the starting cell type, but, during the process of reprogramming, change their binding sites and end up binding different sites in the reprogrammed cell 100, 101. By contrast, iN‐conversion TFs are typically on‐target TFs, like Ascl1, Ngn2, and NeuroD1, as they bind specific chromatin regions to induce transdifferentiation and stay at those sites also in the converted iNs, unless turned off in doxycycline‐inducible systems 24, 27, 33, 35. As iN conversion is more efficient than iPSC reprogramming by orders of magnitudes, the difference between off‐target iPSC reprogramming and on‐target iN conversion has been suggested to underlie the huge difference in reprogramming efficiency. Still, due to epigenetic differences, not every on‐target pioneer TF is equally potent in every starting cell type, as, for example, Ascl1 is able to induce conversion of fibroblasts, but not keratinocytes, into iNs 24, 30. This has been somewhat surprising, since the overall less efficient iPSC reprogramming TFs are known to equally reprogram a broad variety of somatic cells into pluripotency 102. This is explained by the fact that on‐target pioneer factors require a more specific epigenetic signature in order to bind to closed chromatin and preexisting histone modifications like a trivalent chromatin state of H3K4me1/H3K27ac/H3K9me3 in fibroblasts, which are one reason why Ascl1 can induce direct iN conversion only in fibroblasts (Fig. 1D) 24.

In fibroblast‐to‐iN conversion, Ascl1 exerts its function already a few hours after induction, acting as a transcriptional activator inducing neuronal‐ and muscle‐related gene expression 32. Until day 5, Ascl1 alone is responsible for 80% of chromatin changes occurring during the whole reprogramming process, leading to an upregulation of genes involved in neuronal processes, neuronal network formation, and early genes of neuronal maturation 32. Downstream of Ascl1, TFs like Zfp238 additionally influence the expression of genes involved in chromatin remodeling, including methylases 24, 103. Some iN protocols that are not quite efficient can result in cells that lack epigenetic marks of mature neurons 48, 51. Modifying the epigenetic landscape, for instance, with the help of small molecules like forskolin, or overexpressing additional factors like Brn2 and Myt1l, aids to efficiently generate mature neurons 33, 48, 51. The importance of methylation remodeling in neuronal maturation is further supported by the inability to generate mature neurons upon knockout of genes involved in histone methylation, which control accessibility to genes involved in synapse formation and neuronal function 103. Another milestone achievement in epigenetics has been mouse microglia‐to‐iN conversion 27. Here, the pioneer TF NeuroD1 binds unmethylated CpG‐rich regions and rearranges the bivalent H3K4me3/H3K27me3 state toward a monovalent H3K4me3 state during conversion. Interestingly, the secondary TFs Prdm8, Bhlhe22, and Brn2, which are direct target genes of NeuroD1 in microglia, can also facilitate microglia‐to‐iN conversion by either inducing neuronal gene expression (Brn2) or repressing microglial genes (Bhlhe22, Prdm8) 27. Further, cell type‐specific epigenetic characteristics that pose a hurdle for direct conversion from keratinocytes also hinder conversion of human cardiomyocytes, which have been shown to only marginally convert into neurons with BAM/NeuroD1 104. Similarly, the development of robust protocols for iN generation from stored human blood samples has been as much anticipated as it has been challenging. In a proof of principle, adult human peripheral blood mononuclear cells have been successfully converted into functional iNs using electroporation of BAM/NeuroD1 91, which is a less efficient, but also a less immunogenic TF delivery method than viruses. Further, albeit the use of well‐selected small‐molecular boosters and coculture with primary mouse glial cells, blood‐to‐iN conversion in comparison still faces low efficiencies 91.

## You are what you eat: Metabolic hallmarks of iN conversion

Originally regarded more as a mere characteristic of neurons, a growing body of evidence now suggests a central role for metabolic regulation in differentiation and direct iN conversion 95, 105. Following the initiation of neuronal transcriptional programs, converting cells are forced to adapt neuronal metabolism (Fig. 1E) 9, 106, 107. Compared to fibroblasts or astrocytes, neurons rely heavily on oxidative phosphorylation to meet their high demand of energy 108, 109, 110. The metabolic switch from aerobic glycolysis to neuron‐specific oxidative phosphorylation is crucial for the generation of neurons. Inhibiting oxidative phosphorylation or overexpressing glycolysis genes such LDHA or HK2 results in diminished neuronal differentiation 65. While during neural stem cell differentiation cells can slowly adapt to the metabolic switch, direct conversion forces fibroblasts to rapidly switch to oxidative phosphorylation, resulting in increased oxidative stress 64. This observation has led to the view that the metabolic switch represents a major roadblock in direct conversion as it triggers cell death in more than 80% of transgene‐induced cells 64. This vast amount of stress‐induced cell death during conversion could be prevented by either overexpressing anti‐apoptotic protein Bcl‐2 or adding molecules implicated in the anti‐oxidative stress response, which further resulted in a more effective and faster conversion from astrocytes 64. In consistence with the view that the metabolic shift from fibroblasts to neurons represents a major roadblock for successful conversion, chemical derepression of oxidative phosphorylation using the Hif‐1α translation inhibitor KC7F2 during conversion leads to increased mitochondrial membrane potentials and more robust and efficient iN conversion from adult and old human donor fibroblasts 41.

## Updating our criteria to define neurons

‘Well well well, so you want to be a *real* neuron…’. Since the first neurons were generated from human embryonic stem cells, an evergreen question has been if obtained cells should be called neurons, or if they should rather be described as neuronal‐like cells. While some argue that human stem cell‐derived neurons should be called a neuron once they meet a set of electrophysiological characteristics, others suggest to rather call them neuronal‐like cells to avoid confusion with primary neurons 111. While the question of naming seems to be merely of interest to nomenclature aficionados, it is out of the question that it is vital to possess a wide range of relevant criteria, as well as a well‐stocked toolbox to measure a cell’s neuronal identity. While reprogrammed neurons might always be distinguishable from *in vivo*‐born cells, recent work has demonstrated that the environment of cells appears to be more important for cellular identity than the origin of cells, indicating that cell identity is plastic and highly environment‐dependent 112, 113. Further, unlike for most other human cell types, primary human neuronal cultures of defined brain regions are not available for direct comparison, which further complicates the definition of gold standard criteria. By any means, the value of cell models should be measured by how well they perform their respective tasks, and these tasks might range from teaching us more about a complex disease, or how well they integrate into a neuronal circuit following transplantation 6, 33, 34, 35, 36, 37, 38, 114, 115.

With the establishment of (epi)genomic sequencing and epigenetic array technologies, single‐cell technologies, and new insights into the complexity of neurological diseases, the historic neuron‐electrophysiology‐centric view on neuronal identity, function, and disease is fading 35, 51, 69, 116. Thus, also a broadened set of criteria on how to characterize a neuron using such new omics technologies in addition to the classical cell biological and electrophysiological measures is uprising (Fig. 1F). Still, the most common way to assess neuronal cultures is to perform immunocytochemical analysis with markers like β‐tubulin, MAP2, DCX, synapsin I, or tau and to assess neuronal morphology according to soma size and number and length of dendrites, as it is standard practice in the stem cell field 15, 41, 81, 97, 117. Additionally, electrophysiological analysis has proven valuable to define functional neurons, as neurons have very specific electrophysiological features like hyperpolarized membrane potential and spontaneous and triggered action potentials 32, 46, 49. For many studies that are not neuronal function‐centered, Ca^2+^ imaging and multielectrode arrays can be used as surrogates for electrophysiology and can further deliver insights into neuronal network activity *in vitro* 20, 21, 119, 120. In this regard, iNs have been shown to express neuronal markers, show mature neuronal morphology, and possess both spontaneous and induced postsynaptic currents 15, 57, 81, 118, 119. Differentiating neurons in the developing brain and in iPSC differentiation pass through stages that specifically allow them to participate in functional circuits 121, while iNs skip these stages. This raises the question as to whether directly converted neurons have the neuron‐specific ability to create and integrate into a neuronal network. It has been shown that iNs, despite following a different developmental path than neurons in the brain, can survive and differentiate after transplantation into the mouse brain or cultivation on organotypic brain slices 57, 81. Outstandingly, iNs are capable of long‐distance axonal outgrowth, following axonal growth cues specific for the neuronal subtype and connecting with target brain regions, providing evidence of a stable conversion of cells into functional, fully differentiated neurons 57.

While classical cell biological and electrophysiological criteria are a gold standard for characterizing such cells in culture, and transplantation experiments have a strong relevance and broad implications, they do not tell us much about more general measures that define cell identity. Neurons do not only have unique electrical properties, but also have unique epigenetic, proteomic, metabolic, and other characteristics that might be equally important for the roles they play in the brain. While marker expression and electrophysiological characteristics often correlate with cell identity, they are considered to be downstream of epigenetic and proteomic changes and likely follow earlier pathogenic events in neurological diseases. Immunocytochemical data are further quite variable between experiments, antibody batches, and laboratories, thus not well‐quantifiable, low‐dimensional, and thus less ideal to be directly compared by statistically powerful (meta‐)analytical means. Conversely, gene expression changes related to synapse formation, voltage‐gated potassium channels, or mitochondrial oxidative phosphorylation critically define mature neuronal identity 122, and transcriptomes of single human iPSC‐derived neurons can clearly predict neuronal functionality 123. As epigenomic, transcriptomic, proteomic, and metabolic profiles of human brain samples are emerging, we can now use these data and map iN and iPSC‐derived data to them. A prime source for these data is transcriptome databases of the adult and developing human brain 124, 125, 126, which have been exploited by stem cell scientists to learn more about the underlying identity of cultured neurons. For example, Stein *et al*. 127 developed a machine learning approach that can help to identify the developmental maturity and regional identity of *in vitro* models, which has detected marked differences between laboratories and differentiation protocols. Nayler et al. 128 have compared the transcriptomes of iPSC‐derived cerebellar neurons to the Allen Brain Atlas to demonstrate that their cells are transcriptionally similar to discrete regions of the human cerebellum at the second trimester of development. Camp et al. 129 compared single‐cell transcriptome data derived from iPSC cerebral organoids to *in vivo* data and found that the cells recapitulate gene expression trajectories that correspond specifically to human fetal neocortical development. This concept was later extended toward new insights into cellular diversity, once also *in vivo* single‐cell data of the developing human brain became available 130, 131, 132, 133. In the iN field, Vadodaria *et al*. 35 used a transcriptome comparison to confirm serotonergic iN identity, as they found a high similarity of serotonergic iNs to the transcriptomes of the human raphe nucleus. While these types of analysis should not be considered to fully replace marker expression and functional analyses, they are highly complementary and clearly add additional value.

Further, biological or epigenetic age can be well measured using the DNA methylation profile exploiting a growing number of epigenetic clock algorithms 134, 135, 136. The original epigenetic clock used the methylation values of 353 specific CpG loci to calculate the age of a human sample, and Huh *et al*. have employed this tool to demonstrate for the first time that iNs from adult donors are indeed (epigenetically) adult cells, while iPSCs and their derivatives are typically rejuvenated into prenatal epigenetic ages 134, 137, 138. Further along epigenomic criteria, and in contrast to CpG methylations, non‐CpG methylation (mCH) marks are a typical characteristic of neurons 139, 140. In mature neurons, mCH marks are accumulated at particularly high levels, which was not observed in any other tissue before 139. mCH marks play an essential role in cell type‐specific fine‐tuning of transcription, being responsible for dynamic expression patterns during early differentiation, and later represent stable repressors in mature neurons 51. These methylation marks are strong indicators of mature neurons and may further contribute to the vast diversity of neuronal subtypes in the human brain 140, 141. Luo *et al*. recently showed that in contrast to iPSC‐derived neurons, BAM‐based iNs are the first cellular neuronal model system displaying this epigenetic hallmark of mature adult neurons 51, 142, 143. Overall, as neurons are generally defined by their marker expression and electrophysiological properties, the ascent of next‐generation sequencing methods has allowed to carefully characterize iNs according to their gene expression profile and epigenetic landscape, representing more detailed and reliable validations. We expect such criteria to become more and more popular, extending our knowledge of cell identity, and that they will eventually largely replace classical means of neuronal characterization.

## The direct path to the end: Specifics of iN and their consequences for disease modeling

**Figure 2:**
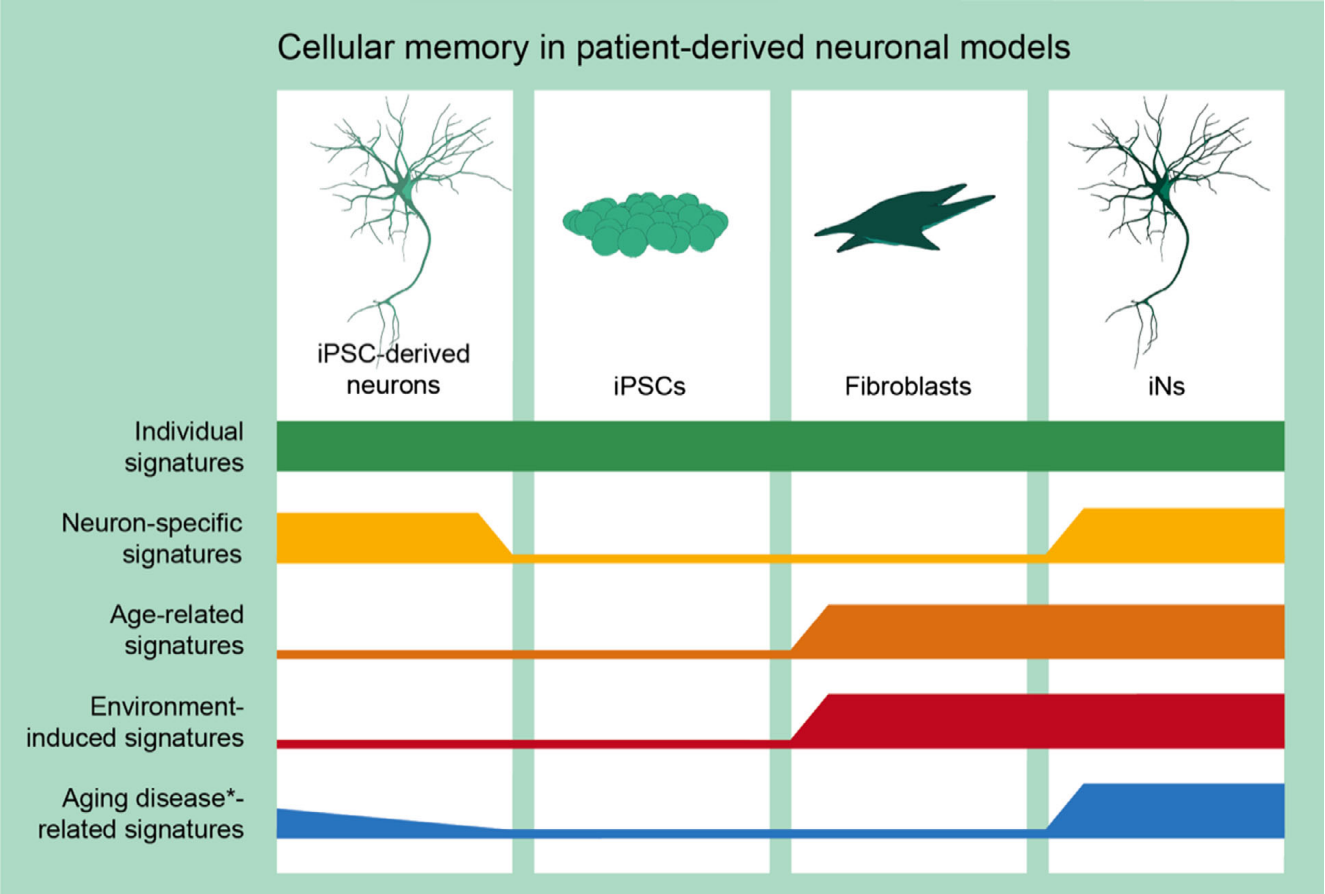
Human patient‐specific models are representative of the individual’s genetic and epigenetic signatures. These individual signatures may vary between cell types, but are present in fibroblasts, iPSCs, iPSC‐derived neurons, and iNs likewise. Neuron‐specific signatures are present only in iPSC‐derived neurons and iNs, but not in fibroblasts or iPSCs. Contrary to iPSC reprogramming, direct iN conversion preserves signatures of donor age and likely also captures environment‐induced signatures, which might or might not be relevant for the disease model. In the context of a multiple hit theory for age‐related diseases, it appears conceivable that features of such diseases might only emerge in iN models and not in iPSC‐based models, because they require all the individual signatures, neuron‐specific signatures, and age‐related signatures to unfold in the cells. Artificial induction of age in iPSC‐based models might help to elicit such features also in a rejuvenated context.

It is primarily due to the inaccessibility of live human brain tissue that most studies on complex age‐related neurodegenerative disorders have primarily relied on transgenic animal models that, while yielding important insights, have also revealed limitations regarding transferability to human physiology. *In vitro* generation of patient‐specific neurons from iPSCs for modeling diseases of the brain has evolved into an integral part of neuroscience 144, 145, 146, and employing human iPSC technology to investigate aspects of age‐related neurodegenerative diseases in a patient‐specific genetic context at a cellular level has yielded important human neuron‐specific insights 147, 148, 149, 150. However, iPSC‐based studies could not shed much new light on the causes of sporadic age‐related diseases, because iPSC reprogramming is known to reset the epigenetic state of the cell and erases most of the epigenetic memory, including those that stem from potentially important environmental influences (Fig. 2) 10, 151, 152, 153, 154, 155, 156. The rejuvenation effect of iPSC reprogramming is a major drawback when attempting to model late‐onset diseases 17, 157, 158, and artificial induction of the factor age by overexpressing progerin 151, shortening of telomeres 159, or exposing cells to age‐related stressors 12, 160 is an upcoming and widely used strategy to elicit a relevant phenotype in iPSC models for neurodegenerative diseases 12, 148, 161, 162, 163. Direct iN conversion of human fibroblasts from elderly human patients and healthy donors into iNs circumvents this issue and has attracted broad attention. Old human donor‐derived iNs show stark transcriptomic signatures of aging, as well as nuclear pore and transport‐associated aging 10, 164, metabolic and mitochondrial aging 9, 137, epigenetic aging 137, and other aspects of cellular aging (Fig. 2) 12, 17, 157. Human iNs thus stand out as a highly attractive patient‐specific model system for age‐related neurological diseases and thus significantly extend the possibilities offered by iPSCs. For example, while iPSC‐derived neurons from genetically defined amyotrophic lateral sclerosis (ALS) or Huntington’s disease (HD) patients are a promising model for investigating these devastating diseases 165, 166, 167, both HD and ALS show an adult‐onset pathology that is also influenced by aging. Because iPSC‐derived neurons have a fetal developmental identity and because age‐related cellular defects are erased, the study of a disease‐related pathology in these cells is challenging and external stressors are typically used to provoke phenotypes 167, 168, 169. iNs, however, bypass these stages of fetal development, preserve disease‐relevant defects associated with cellular aging, and recapitulate disease‐related phenotypes including morphological, survival, and functional defects (Fig. 2) 77. The key advantage of such cellular models over postmortem brain tissue is that they permit to experimentally test cause–consequence relationships via functional studies, and to evaluate molecular intervention with potential drug‐like molecules.

Importantly, the rejuvenating effect of iPSC reprogramming should mostly be regarded as an additional advantage, because comparing phenotypically young and phenotypically old neurons from the same donor (isogenic rejuvenated control) can help to reduce donor‐specific bias and allows assessing the contribution of age to the disease phenotype. Although it is a new concept, harnessing the power of combining iPSC differentiation and direct iN conversion has already been published. Tang *et al*. 157 have generated induced motor neurons, as well as iPSC‐derived motor neurons from three young (0–3 years) and three old (53–71 years) healthy donors, as well as from four familial ALS patients carrying SOD1 or FUS mutations. As expected, the rejuvenated iPSC‐derived motor neurons did not show age‐related differences, while iN‐converted, age‐equivalent induced motor neurons showed nuclear envelope defects, increased signs of DNA damage/repair (γH2AX), and age‐dependent deterioration of the cellular aging markers Lap2α, H3K9me3, and HP1γ, which are likely downstream of a defective nuclear envelope 151. While this study did not assess any age‐related differences between patient‐ and control‐derived induced motor neurons, an impressive study by Victor *et al*. 7 demonstrated the importance of modeling old age as a disease‐relevant factor in a model consisting of both iPSC‐derived and fibroblast‐derived medium spiny iNs. Mutated huntingtin protein spontaneously aggregated in HD iNs, but not in control iNs, HD fibroblasts, or HD iPSC‐derived neurons. In consistence with the observation that HD iPSC‐derived neurons need external stressors to display this disease phenotype, the authors showed that an age‐related collapse in proteostasis triggered huntingtin aggregation in an age‐ and repeat‐length‐dependent manner 7. In the context of a multiple hit theory of age‐related diseases, where at least one essential ‘hit’ is old age and other ‘hits’ are of either genomic or environmental nature, these data suggest two major contributors for HD, namely age and repeat length, that together determine onset and severity of the pathology. However, in addition to age, it appears likely that also other epigenetic signatures of the donor that relate to environmental signals are also preserved in iNs. For the resulting iN model, such environment‐induced signatures might be irrelevant or even artifact‐causing (e.g., if they relate to sun exposure of the skin), but they probably also involve important disease‐related signatures that might be encoded within fibroblasts, but stay without stark transcriptional consequences until they are brought into a neuronal context via iN (Fig. 2).

While most studies imply that iN conversion is particularly useful for modeling age‐associated diseases, direct reprogramming approach is not always the method of choice for disease modeling, especially not when it comes to developmental diseases such as autism spectrum disorder. Schafer *et al*. have used iPSC differentiation and iN conversion starting from iPSCs in parallel, and only iPSC differentiation revealed a disease‐related phenotype, while iN conversion did not. Specifically, the study explored developmental transcriptomic and epigenomic trajectories in autism spectrum disorder using classical neural differentiation and cerebral organoids. As opposed to the most widely believed assumption that the first disease phenotypes emerge in immature neurons, they found heterochronic trajectories in patient cells that were already epigenetically primed for acceleration at the neural stem cell stage 16. To pinpoint the origin of this phenotype to neural stem cells, the authors also made use of direct Ngn2‐based iN conversion starting from iPSCs to skip (jump over) the neural stem cell stage and consequently found no autism spectrum disorder‐related neuronal phenotypes in iNs.

There are probably more meaningful conceptual differences between iN‐based and iPSC‐derived models that only wait to be explored and that have the potential to significantly improve *in vitro* disease modeling in the future. For example, iPSC derivatives have been shown to resemble fetal stages of brain development, and it remains an open question to what extent iNs from fetal, neonatal, and adult donor fibroblasts would resemble neurons of the according developmental stages. These questions must be addressed with state‐of‐the‐art tools for defining and comparing cell identities. A fetal identity of neurons after iPSC differentiation, however, does not implicate a functionally immature cellular phenotype, as both directly converted neurons and iPSC‐derived neurons can show mature neuronal markers and features 10, 81, 157, 170.

Additionally, iNs exhibit various technical advantages as well as critical disadvantages compared to iPSC reprogramming, which have been discussed more in detail elsewhere 17. One of the major limiting factors of direct conversion models is that the starting material is finite, and no expandable stem cell stages are involved in the process. As a result, iN cell numbers are low and the ability to scale up the system (e.g., through immortalization of fibroblasts) harbors risks of introducing artifacts that might invalidate their use as a model for aging. Thus, material‐intensive technologies or big screens are challenging with iNs 171. One practical and obvious advantage of iNs is that the procedure is faster, easier, and cheaper than iPSC reprogramming and differentiation, and many iN papers have stood out by comparatively large numbers of human donors 7, 9, 10, 137. Biological variability between human samples and cell lines of different genetic backgrounds has been identified as a major challenge in human iPSC‐based disease modeling 60. While more variability between humans than inbred mice had definitely to be expected, variability has apparently caught the disease modeling field by surprise and has caused doubt about the technology. Here, iNs provide the opportunity to advance the reproducibility and relevance of human disease modeling studies, as higher patient and control numbers can be used, thereby making the application of powerful statistical/bioinformatical tools for data analysis useful 172. iNs further do not only represent interindividual variability, but also the fibroblast culture of each patient exhibits a certain degree of heterogeneity (mosaicism) compared to the clonal identity of iPSCs. This difference might be regarded as a disadvantage, for instance, for the generation of isogenic control lines, or as an advantage when cell mosaicism may play a role, such as in aging or psychiatric conditions 173.

## Conclusion

Direct iN conversion offers a valuable addition to iPSCs to study the fundamentals of cell identity, investigate human neuronal function, and model neurological diseases, and as a new strategy for *in vivo* cell replacement therapies. Unlike iPSC‐based differentiation to neurons, iNs circumvent the known paths of neurodevelopment, and we have started to better understand the mechanics of this process in the recent years. The unique characteristics of iNs already let them stand out as a valuable tool for many applications. Among the best‐known phenomena is the age‐preserving characteristic of iNs, which makes them a useful complement to iPSC models of brain aging and age‐related diseases. Today is only the dawn of this technology, and the development of more direct conversion protocols and applications can be expected in the near future. Application of iN technology profits from the rapid developments in the iPSC field, and iNs already leave their mark in the current process of redefining how we think about cell fate, neuronal identity, and possibilities to deconstruct and reconstruct the inner workings of the human brain.
